# Bacterial identification using MALDI-TOF mass spectrometry in positive blood cultures: A pilot study

**DOI:** 10.14440/jbm.2025.0119

**Published:** 2025-03-12

**Authors:** Smriti Srivastava, Akshay Shankar, Neha Sharad, Aparna Ningombam, Kamran Farooque, Purva Mathur

**Affiliations:** 1 Department of Microbiology, Jai Prakash Narayan Apex Trauma Centre, All India Institute of Medical Sciences, New Delhi 110029, India; 2 Department of Lab Medicine, Jai Prakash Narayan Apex Trauma Centre, All India Institute of Medical Sciences, New Delhi 110029, India; 3 Department of Orthopedics, Jai Prakash Narayan Apex Trauma Centre, All India Institute of Medical Sciences, New Delhi 110029, India

**Keywords:** MALDI-TOF mass spectrometry, Blood culture, Bloodstream infection, Rapid diagnosis

## Abstract

**Background:**

Early pathogen identification in the bloodstream has long been a key focus for microbiologists and clinicians, given its crucial role in patient management. Matrix-assisted laser desorption ionization-time of flight mass spectrometry has emerged as a valuable tool for the direct and rapid identification of organisms from positive blood cultures.

**Objective:**

This study aimed to evaluate the accuracy, productivity, and feasibility of two methods for the rapid detection of bloodstream infections.

**Methods:**

Two methods were employed in this study: One based on differential centrifugation and the other using a lysis buffer.

**Results:**

The addition of a lysis buffer, sodium dodecyl sulfate (SDS), to the blood culture broth resulted in the identification of a greater number of microorganisms (*Acinetobacter baumannii, Pseudomonas aeruginosa*, and *Klebsiella pneumonia*).

**Conclusion:**

The application of SDS into culture broths is user-friendly and can be easily integrated into routine blood culture processing, allowing for species-level identification within hours of a positive BacT/ALERT signal.

## 1. Introduction

Rapid diagnosis of bloodstream infections (BSIs) is crucial for patient survival and remains a significant focus for microbiologists. The goal is to develop assays with faster turnaround times and higher sensitivity for detection of bacteremia. Over the past few decades, microbiological culturing has advanced toward automated, continuously monitored, signal-based blood culture techniques.^1^ Integrating these systems with rapid microbial identification systems, such as matrix-assisted laser desorption ionization-time of flight mass spectrometry (MALDI-TOF MS), can significantly reduce reporting time. Moreover, MALDI-TOF MS is gaining popularity in clinical microbiology laboratories due to its ability to facilitate early identification, ease of operation, and relatively low running costs.^2^ A notable application of MALDI-TOF MS is its ability to identify pathogens directly from positive blood cultures, addressing the traditionally longer reporting time associated with blood culture reporting compared to other samples. Rapid identification of bacteremia is among the most critical microbial diagnostic emergencies.^3^ Other bacterial identification methods, such as nanoparticle enrichment on bacterial cells through MALDI-TOF MS and biochips for bacterial cell analysis, can also be used for pathogen identification.^4^-^6^ However, there are two key considerations when identifying organisms in positive blood cultures using MALDI-TOF MS. First, the limit of detection is 10^5^ colony-forming units (CFU)/spot for bacteria and 10^4^ CFU/spot for yeast, which requires concentrating the bacteria or yeast from samples.^7^,^8^ Second, various components in the blood culture broth, such as blood cells and the culture media *per se*, must be removed using either physical methods (such as differential centrifugation) or alternative methods, such as clot activation or the use of lysis buffer. In this study, we evaluated two different methods used to identify pathogens directly from blood samples using MALDI-TOF MS. We explored their effectiveness in the rapid detection of BSIs, discussed the integration of MALDI-TOF MS into the blood culture workflow, and highlighted the promising array of pathogens identified.

## 2. Materials and methods

The study was conducted at the Microbiology Unit of a Level-1 Trauma Center in a northern Indian tertiary hospital. Trauma care hospitals are dynamic emergency centers primarily treating young trauma victims, and the majority of BSIs in these patients are healthcare-associated. In our facility, blood culturing is performed using the BacT/Alert (BioMérieux, France) culture system. Isolate identification follows the subculture of signal-positive bottles onto blood agar (ready-to-use plates procured from BD, USA) and MacConkey agar (BD, USA), with subsequent isolated pathogen identification using Vitek-2 (BioMérieux, France) and/or MALDI-TOFMS (Vitek MS, BioMérieux, France). Preliminary Gram-stain findings from positive blood culture bottles are promptly communicated; however, the turnaround time for final culture reports is 3 days.

In this study, we evaluated two different protocols for the identification of organisms directly from a signal-positive bottle using MALDI-TOF MS.^9^,^10^ Initially, we explored three methods: (i) differential centrifugation, (ii) lysis by 10% sodium dodecyl sulfate (SDS, Sisco Research Laboratories Pvt. Ltd, India), and (ii) the use of a clot activator tube. However, the clot-activator tube method resulted in more failures and required a larger broth volume, leading to its discontinuation. Consequently, we proceeded to evaluate the remaining two methods (Method 1 and Method 2).

### 2.1. Method 1: Differential centrifugation method

Two mL of broth was drawn from the positive-flagged blood culture bottles into 2 mL centrifuge tubes (Eppendorf, Germany). The first centrifugation was performed at 400 × g for 10 min. The supernatant was collected, and the cellular debris was discarded. The second centrifugation was conducted at 17,000 × g for 5 min. The resulting pellet was washed twice with 1.5 mL of distilled water (17,000 × g for 3 min) to further remove blood components from the bacterial pellet. After drying the pellet for 10 min at room temperature, approximately 50 μL of semi-solid pellet was obtained. A volume of 1 μL of this pellet was then inoculated onto a Vitek MS (BioMérieux, France), followed by sequential overlaying with 0.5 mL of formic acid and 1 μL of matrix solution (α-cyano-4-hydroxycinnamic acid [CHCA]). Both formic acid and CHCA were procured ready-to-use from BioMérieux (France). After complete drying, the slide was inserted into the instrument. Vitek MS uses a standard strain of *Escherichia coli* (ATCC® 8739™) as an internal standard, applied at the central well among the set of 16 wells. The test culture was applied uniformly onto the wells in duplicate. After scanning the slide using the barcode scanner provided with the instrument, the slide was inserted into the instrument slot. The slide was then interrogated using a fixed focus, 337 nm nitrogen laser at the rate of 50 laser shots per second. Proteins were detected on a sensor, and a spectrum was generated, which was classified using a proprietary advanced classifier. These spectra were then compared to the database to identify genera and species.

### 2.2. Method 2: Lysis using 10% SDS

**Figure 1:**
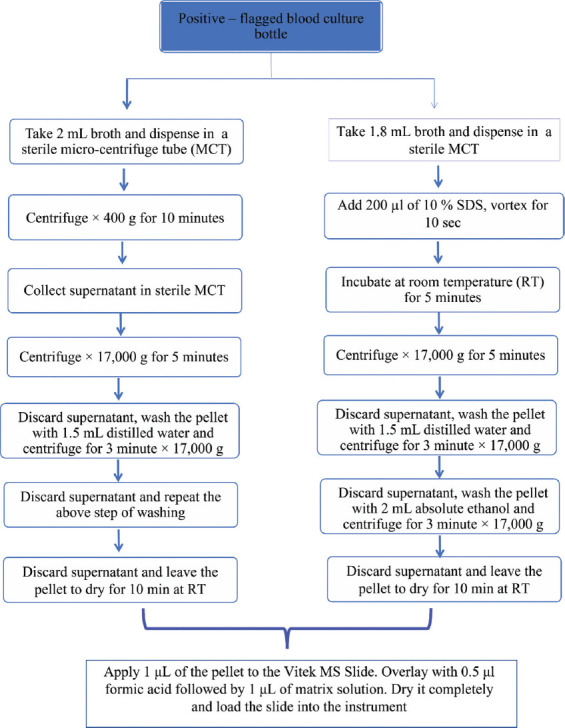
Methodological flowchart. Microbial identification from Method 1 (left) and Method 2 (right) for comparative study Abbreviation: SDS: Sodium dodecyl sulfate.

Two mL of broth was drawn from the positive blood culture bottle, with 1.8 mL transferred to a 2 mL microcentrifuge tube, followed by the addition of 200 μL of 10% SDS solution. The resulting mixture was vortexed for approximately 10 s and then incubated at room temperature for 5 min. Next, centrifugation was performed at 17,000 × g for 5 min. The supernatant was removed, and the pellet was resuspended in distilled water before undergoing another centrifugation at 17,000 × g for 3 min. The supernatant was again discarded, and the pellet was resuspended in 2 mL of 70 % ethanol, followed by a final centrifugation for 3 min at 17,000 × g. The pellet was dried for 10 min at room temperature. From the resulting semi-solid pellet of approximately 50 μL, 1 μL was then applied for MALDI-TOF MS analysis, following the same procedure as in Method 1 (Section 2.1.). Both methods are outlined in Figure 1.

The identification results from both methods were compared to the standard procedure in our laboratory, which involves growth on solid media followed by identification of isolates using MALDI-TOF MS. Growth on solid media such as blood agar was used. An isolated colony was selected and carefully applied to the well on the Vitek MS slide, followed by the addition of a matrix, replicating the Vitek MS process.

Before the commencement of the study, the operators were thoroughly briefed on the procedures. For this purpose, a blood culture bottle was seeded with 10 CFU of *E. coli* ATCC 25922 and incubated in the BacT/Alert system. Once the bottle was flagged positive by the system, it was used for testing with both methods. However, our primary aim was to evaluate the performance of both methods in real clinical scenarios, where pre-analytical variables, such as organism load in patient samples, blood volume, and duration before incubation, may differ from controlled laboratory conditions. Hence, pre-fabricated samples were not used further in the study.

### 2.3. Statistical analysis

GraphPad (GraphPad Software Inc) and MS Excel (Microsoft Inc.) were used for statistical analysis. Categorical variables were presented as numbers (%) and compared by the χ^2^ test.

## 3. Results

A total of 20 patient samples were subjected to both protocols, and the results are shown in Table 1. The data presented in the table are pre-clinical. The agreement between Method 1 or Method 2 and the standard protocol was found to be 50.0% and 80.0%, respectively (χ^2^ [1, n = 20] = 2.7473, *p* = 0.097422). No incorrect identification was found.

**Table 1:** Direct identification methods versus standard laboratory procedure

Sample no.	Identification using Method 1	Identification using Method 2	Identification after growth in culture (standard protocol)
1	Yes	Yes	*A. baumannii*
2	Yes	No	*K. pneumonia*
3	No	Yes	*A. baumannii*
4	No	No	*S. maltophila*
5	Yes	No	*K. pneumonia*
6	No	Yes	*Salmonella enteric subsp. enterica*
7	No	No	*P. stuartii*
8	Yes	Yes	*P. stuartii*
9	No	Yes	*A. baumannii*
10	Yes	Yes	*P. aeruginosa*
11	No	Yes	*K. pneumonia*
12	Yes	Yes	*P. aeruginosa*
13	Yes	Yes	*S. maltophila*
14	Yes	Yes	*Serratia marcescens*
15	No	Yes	*K. pneumonia e*
16	No	Yes	*Staphylococcus hominis*
17	Yes	Yes	*K. pneumonia e*
18	No	Yes	*Alcaligenes faecalis*
19	No	Yes	*Staphylococcus aureus*
20	Yes	Yes	*K. pneumonia*

Abbreviations: *A. baumannii: Acinetobacter baumannii; K. pneumonia: Klebsiella pneumonia; S. maltophila: Stenotrophomonas maltophila; P. stuartii: Providencia stuartii; P. aeruginosa: Pseudomonas aeruginosa.*

**Figure 2:**
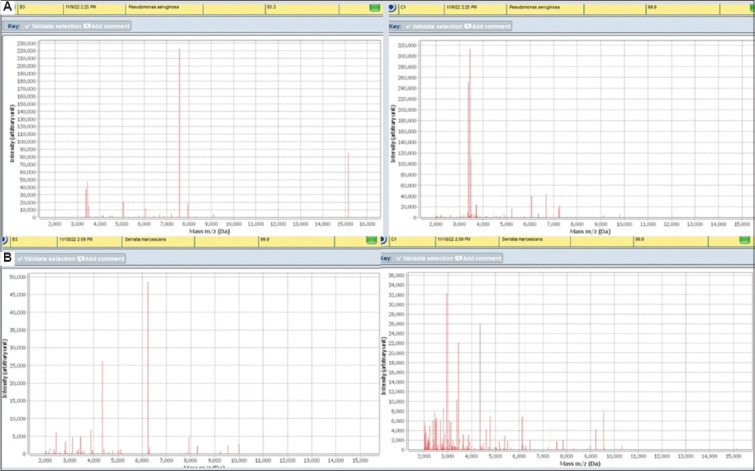
Mass-to-charge ratio (m/z) spectra from both methods. (A) Method 1 demonstrates m/z peaks around 6,000 – 8,000 Daltons, while (B) Method 2 presents m/z peaks around 2,000 – 3,000 Daltons

The spectral output from both methods differed, regardless of the result. Method 1 demonstrated a lower intensity of mass-to-charge ratio (m/z) peaks around 6,000 – 8,000 Daltons, whereas Method 2 exhibited a higher intensity of m/z peaks around 2,000 – 3,000 Daltons, as shown in Figure 2.

We compared the ease of performing the two methods between two operators by asking them to rate each step as “easy,” “not so easy,” and “difficult/tedious,” assigning difficulty scores of 1, 2, and 3, respectively (with the best possible score being 4). This evaluation was based on four parameters: Ease of withdrawing blood from the bottle, number of centrifugation steps, supernatant collection, and pellet re-suspension after discarding the supernatant. These parameters were graded on a scale of 1 (easy), 2 (not so easy), and 3 (difficult/tedious) by two operators who performed the methods independently. A comparison of these aspects for both methods is summarized in Table 2.

**Table 2:** Comparison between the two methods

Parameters	Method 1		Method 2	
Time taken^a^	35 – 40 min		30 – 35 min	

**Ease of performing**	**Operator 1**	**Operator 2**	**Operator 1**	**Operator 2**

Withdrawing blood from the bottle	1	1	1	1
Too many centrifugation steps	2	1	1	1
Collecting the supernatant	1	1	1	2
Resuspending the pellet after discarding the supernatant	1	1	1	1
Overall score	5	4	4	5
Additional consumables required	Syringe and needle – 2 mL, 2 mL centrifuge tubes (2), formic acid (0.5 µL), and CHCA (1 µL)		Syringe and needle – 2 mL, 2 mL centrifuge tubes (1), absolute ethanol (2 ml), formic acid (0.5 µL), CHCA (1 mL), and SDS^b^ (200 µL)	

Notes:

a Time taken does not include the run time for MALDI-TOF MS, which also depends on other wells on the slide.

b This is equivalent to 20 mg SDS per reaction.

Abbreviations: CHCA:α-cyano-4-hydroxycinnamic acid; MALDI-TOF MS: Matrix-assisted laser desorption ionization-time of flight mass spectrometry; SDS: Sodium dodecyl sulfate.

**Figure 3:**
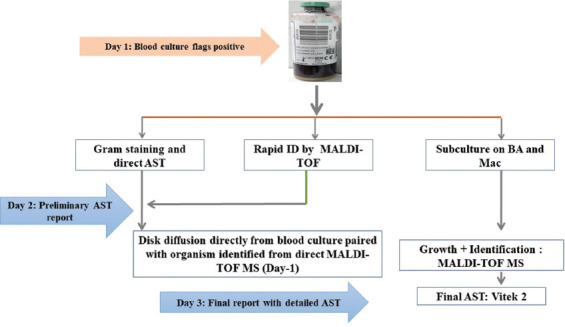
Blood-culture workflow for microbial identification to antibiotic susceptibility test Abbreviations: AST: Antibiotic susceptibility test; BA: Blood agar; ID: Identification; Mac: MacConkey agar; MALDI-TOF MS: Matrix-assisted laser desorption ionization-time of flight mass spectrometry.

Since March 2023, we have incorporated direct MALDI-TOF MS from positive blood culture bottles into our routine sample processing protocol. This allows results to be communicated to the treating physician, enabling modifications to antibiotic therapy, if needed, based on our 6-monthly antibiograms. Further refinement is achieved through preliminary antibiotic susceptibility testing (AST) directly from broth, followed by a final AST from growth on solid media, as illustrated in Figure 3. Using this protocol, we identified a number of organisms (*n* = 56), including some pathogens with fastidious requirements, such as *Brucella spp*., and critical pathogens, such as *Listeria monocytogenes* and *Candida spp*. A list of these prominent pathogens and their associated antimicrobial resistance profiles is provided in Table 3.

**Table 3:** Antimicrobial resistance profile of the predominant pathogens

Organism	No.	Antimicrobial resistance profile^a^		

		Extended-spectrum beta-lactamases/3^rd^ gen. cephalosporin resistance	Carbapenem resistance	Empirical treatment
*Klebsiella pneumoniae*	16	100% (12/12)	83.3% (10/12)	Drug combinations containing polymyxins or aztreonam
*Pseudomonas aeruginosa*	13	91.66% (11/12)	83.3 (10/12)	Drug combinations containing polymyxins
*Acinetobacter baumannii*	9	100% (7/7)	100% (7/7)	Drug combinations containing sulbactam, minocycline, or tigecycline
*Klebsiella oxytoca*	2	0	0	Cephalosporins

Note:

a Antibiotic susceptibility tests were only performed on fresh isolates.

The remaining organisms consisted of coagulase-negative *Staphylococci* (*n* = 5), *Mycobacterium genavense* (*n* = 2), *Enterobacter cloacae complex, Providencia stuartii, Serratia marcescens, Stenotrophomonas maltophilia, Achromobacterxylosoxidans, Enterococcus faecium, Brucella* spp., *L. monocytogenes*, and *Candida tropicalis* (*n* = 1 for each organism).

## 4. Discussion

Direct identification of organisms from blood culture bottles positively flagged by automated continuous monitoring systems, such as BACTEC (Becton Dickinson Microbiology Systems, United States) and BacT/ALERT (BioMérieux, France), has been in use for over a decade. Both commercially available kits and in-house protocols have been evaluated by clinical microbiology laboratories.^11^-^13^ At present, MALDI-TOF MS is gaining acceptance, and further applications to reduce identification time need to be implemented. This reduction in time would be particularly helpful for the early treatment of sepsis, where appropriate antimicrobial therapy can be lifesaving.^14^,^15^ Once pathogen identification is complete, empirical therapy can be tailored based on local antibiograms. As we move toward infectious diagnostic stewardship alongside antimicrobial stewardship, test results should guide patient management toward precise antimicrobial therapy within a short timeframe. In pursuit of this goal, in 2019, the Indian Council of Medical Research established an Essential Diagnostic Test List for various levels of Indian health-care settings.^16^,^17^ The list outlines the scope of investigations in different tiers of laboratories. Tertiary-level institutes, such as ours, bear the responsibility of formulating and implementing new or experimental protocols.^18^-^20^ Table 3 highlights how timely pathogen identification can optimize antimicrobial therapy, especially in centers with high antibiotic resistance. For instance, at our referral trauma center, the response to commonly used antibiotics is often low, requiring careful treatment based on both acquired and intrinsic bacterial resistance profiles. In addition, several pathogens, such as *Mycobacteria, Brucella*, and *Candida*, which are difficult to identify and treat, take longer time to identify using conventional protocols. While growth on solid media has numerous advantages, detecting these microbes directly from blood culture broth significantly reduces the time in emergency situations such as sepsis.

To reduce the diagnostic delays, we needed a robust, easy-to-perform, and sensitive method for analyzing blood cultures from critically ill patients. Initially, we evaluated three methods, with the third involving the use of a clot activator tube.^21^ However, this method resulted in more acquisition failures and required a larger broth volume, making it inconvenient compared to the other methods. Consequently, we proceeded with the remaining two methods, ultimately implementing the lysis-buffer method. The application of SDS has shown superior results in other studies. For example, a study reported 100% concordant identification to the species level in blood culture bottles spiked with *Candida*.^22^ SDS is an anionic detergent that binds to proteins with high affinity and is commonly employed for cell lysis and protein extraction.^23^ This facilitates better lysis of erythrocytes and the formation of a bacterial pellet free from non-bacterial cellular debris, resulting in fewer acquisition failures. This was apparent from the reduced intensity of m/z peaks around 6,000 – 8,000 Daltons, which are also associated with hemoglobin, low-molecular-weight serum proteins, and casein peptones from the broth.^24^,^25^ We successfully integrated this method into routine practice, thereby shortening the turnaround time for diagnosing BSIs.

## 5. Conclusion

The application of SDS-lysis to blood culture broth facilitates the accurate identification of bacterial pathogens, rendering it suitable to be incorporated into routine diagnostic microbiology laboratories. This procedure significantly reduces the time required for species-level identification by bypassing the need for growth on solid media. The clinical prospects are substantial, and microbiology laboratories employing MALDI-TOF MS should adopt a protocol that is feasible for their specific settings.

A limitation of this study is that it primarily focused on the implementation of the new protocol. Further data are needed to assess how the laboratory outcomes translate into improved patient care. This remains an ongoing long-term objective, aiming to quantify the eventual benefits in patient outcomes.

## Data Availability

Data will be made available on request.
